# Oral anticoagulants in pediatric cardiac practice: A systematic review of the literature

**DOI:** 10.4103/0974-2069.64371

**Published:** 2010

**Authors:** Shreepal Jain, Balu Vaidyanathan

**Affiliations:** Department of Pediatric Cardiology, Amrita Institute of Medical Sciences, Kochi, India

**Keywords:** Oral anticoagulants, pediatric practice, Fontan surgery

## Abstract

Recent advances in the pediatric heart surgery, especially the Fontan procedure, has necessitated an increased use of oral anticoagulants in pediatric cardiac patients. Warfarin is the standard agent used for most pediatric indications, though there are very few randomized control studies in children regarding its use. This review summarizes the current indications and evidence base regarding the use of oral anticoagulants in the pediatric age group.

## INTRODUCTION

The use of oral anticoagulants in children has increased in recent years, parallel with surgical advances for the treatment of univentricular heart, increasing use of prosthetic valves in children and adolescents and rising numbers of children harbouring giant aneurysms following Kawasaki disease. Vitamin K antagonists (VKA, Warfarin and Acenocoumerol) remain the most commonly used oral anticoagulants in children. The first published report of use of Warfarin in children was in 1976, although the drug was in use in the pediatric age group since 1962.[1] Newer oral anticoagulants that act via direct thrombin inhibition (Ximelagatran) or direct Factor Xa inhibition (Rivaroxaban) have superior pharmacokinetics than standard agents. However, these are sparingly used in children and therefore there is only limited available data. This review summarizes the phramacokinetics, indications and current evidence base for use of oral anticoagulants in pediatric cardiac practice.

## MECHANISM OF ACTION OF VITAMIN K ANTAGONISTS

VKA produce their anticoagulant effect by interfering with the cyclic interconversion of vitamin K and its 2,3 epoxide.[2] Vitamin K is a cofactor for the post translational carboxylation of glutamate residues of coagulation proteins (factors II, VII, IX and X). In addition to their anticoagulant effect, the VKA inhibit carboxylation of the regulatory anticoagulant proteins C and S and therefore have the potential to exert a procoagulant effect.

## PHARMACOKINETICS

Warfarin has a high bioavailability and reaches maximal blood concentrations within 90 min of oral administration. The drug is highly protein bound (99%) and is metabolized in the liver through the cytochrome P450 system, with a half-life of 42 h. Ninety-two percent of the drug is excreted through the kidneys, with the remaining being excreted through the biliary tract.[3] Bioavailability and clinical response of warfarin can be modified by several genetic and environmental factors (see the section on drug interactions). Genetic factors that can modify therapeutic response include mutations in the gene coding for the cytochrome P450 2C9 hepatic microsomal enzyme and hereditary resistance to warfarin due to altered affinity to the receptors.[4] Vitamin K-dependent factors are physiologically reduced in the blood of newborn infants, making them more sensitive to these agents.

## DOSAGE AND MONITORING

The standard regime for initiating Warfarin is as described in Table 1.[5] The monitoring of dose is performed by periodic estimation of the prothrombin time-International Normalization Ratio (PT-INR). For most indications, the therapeutic INR range required is between 2.0 and 3.0. For patients with a mechanical prosthetic valve, a higher INR is recommended, depending on the location and type of the prosthetic valve.

**Table 1:** Dosage regimen for initiation and maintenance of Warfarin therapy

A: Loading dose:		
**Initial day I:**	**Days 2-4:**	**Maintenance dose (day 5 and beyond)**
0.2 mg/kg (max 10 mg)	INR 1.1–1.3, repeat loading dose	INR 1.1–1.4, increase dose by 20% of previous dose
0.1 mg/kg, if there is evidence of hepatic dysfunction	INR 1.4–1.9, give 50% of initial loading dose	INR 1.5–1.9, increase dose by 10% of previous dose
	INR 2.0–3.0, give 50% of initial loading dose	INR 2.0–3.0, no change
	INR 3.1–3.5, give 25% of initial loading dose	INR 3.1–3.5, decrease dose by 10% of previous dose
	INR >3.5, hold until <3.5, restart at 50% of previous dose	INR >3.5, hold until <3.5, restart at 20% less than last dose

Younger children generally require relatively higher doses to achieve the desired effect. A recent study of 319 children reported that infants <1 year of age require a daily dose of 0.33 ± 0.2 mg/kg while older children and adolescents require 0.09 ± 0.05 mg/kg to maintain a target INR of 2–3.[6] Patients after Fontan operation require a relatively lower dosage (25% lower) compared to other indications.[6]

## SIDE-EFFECTS

Bleeding is the main side-effect. The risk of major bleeding is 0.5% per patient-year.[6]

The risk of serious bleeding in children receiving VKA for mechanical prosthetic valves is approximately 3.2% per patient-year.[7] The risk increases significantly when the INR is >8. Most cases of bleeding can be treated with vitamin K administration (30 mcg/kg).[8] In life-threatening bleeding complications, fresh frozen plasma should be used. Other adverse effects of warfarin include skin necrosis, gangrene, osteoporosis, fever, hair loss and tracheal calcification.[3]

## CONTRAINDICATIONS

These include hypersensitivity to Warfarin, severe renal or hepatic impairment, cerebral or dissecting aortic aneurysms, active ulceration, severe hypertension, infective endocarditis and pericardial effusion. Pregnancy, in particular the first trimester, is a relative contraindication. Warfarin in a dose of ≤5 mg/day in pregnant patients with mechanical valves provides optimum antithrombotic effect with a low rate of fetal complications.[9]

## INTERACTION WITH DRUGS AND DIETARY AGENTS

The dose of Warfarin needs to be increased when coadministered with anticonvulsants like phenobarbital and carbamazepine. Other drugs interacting with warfarin include aspirin, steroids, nonsteroidal anti-inflammatory agents, alcohol, fluconazole, metronidazole, amoxicillin, rifampicin, chloramphenicol, sulfamethoxazole–trimethoprim combination, etc. Breast milk-fed infants are more sensitive to warfarin as compared to formula-fed infants due to the lower vitamin K content in breast milk. Warfarin effect may be modified by several food items such as liver, broccoli, Brussels sprouts, spinach, coriander, cabbage and other green leafy vegetables. Patients should be advised to maintain constant dietary habits while on warfarin. Further information regarding dietary items and medications affecting warfarin therapy may be found at http://www.drugs.com/coumadin.html.

## PREPARATIONS

Warfarin is available as oral and injectable preparations. The injectable preparation is not widely available in India. Oral tablets are available in strength ranging from 1 to 10 mg. No liquid preparations are available.

## INDICATIONS AND CLINICAL EVIDENCE

The most common indications for use of VKA in pediatric patients include prophylaxis after Fontan surgery, mechanical prosthetic valves and Kawasaki disease with large aneurysms. Other indications include dilated cardiomyopathy with severe left ventricular dysfunction and primary pulmonary hypertension, where the evidence for role of VKA is extrapolated from adult series.[10]

## PROPHYLAXIS AFTER FONTAN SURGERY

Thromboembolism (TE) remains a major cause of morbidity after Fontan operation, with a reported incidence of 3–16% and 3–19% for venous TE and stroke, respectively.[11] There is no consensus in the literature as to the optimal protocol for antithrombotic therapy to prevent such events.[12] In a study by Siepelt *et al*.,[12] the incidence of TE events was 1.1/100 patient-years in the group treated with Heparin followed by warfarin compared to 4.2/100 patient-years in the group with no treatment. American College of Chest Physicians (ACCP) evidence-based clinical practice guidelines recommend Aspirin (1–5 mg/kg/d) or therapeutic unfractionated Heparin followed by VKAs to achieve a target INR of 2.5 (range, 2.0-3.0) for patients undergoing Fontan surgery.[13]

## MECHANICAL PROSTHETIC HEART VALVES

Most of the evidence for anticoagulation after mechanical prosthetic valves is extrapolated from adult studies, with the pediatric data confined to small studies and case reports.[14,15] The incidence of TE in children with mechanical valves is reported to be as high as 68% per patient-year in children who received aspirin and 27% per patient-year for children who received no drug therapy.[16] Addition of VKA reduces the thrombotic complications at an increased risk of bleeding.[14,15] The current recommendations favor use of oral VKA after prosthetic valve replacements to maintain the INR between 2.5 and 3.5, with a higher value for patients with prosthetic valves in the mitral position.[13] For children having thrombotic events while on antithrombotic therapy and in patients where VKA are contraindicated, the ACCP guidelines recommend adding aspirin.[13]

## KAWASAKI DISEASE

Recommendations for antithrombotic therapy in Kawasaki's disease are based on retrospective case studies and extrapolation of data from adult studies of patients with ectactic coronary artery disease.[17] As per the AHA guidelines, oral VKA therapy is recommended to maintain an INR between 2.0 and 2.5 for patients with Kawasaki disease risk level IV (large/giant coronary artery aneurysms or multiple aneurysms without obstruction) and level V (coronary artery obstruction).[17]

## PROPHYLAXIS FOR DILATED CARDIOMYOPATHY

There are no studies of anticoagulant prophylaxis in pediatric patients with dilated cardiomyopathy. In a cross-sectional study of children awaiting cardiac transplantation, 31% of the patients had evidence of acute pulmonary embolism, confirmed by ventilation/perfusion scan or angiography.[18] Based on adult studies, primary prophylaxis with warfarin (target INR 2.5; range, 2.0–3.0) is recommended for patients with severe left ventricular dysfunction.[19]

## IDIOPATHIC PULMONARY ARTERY HYPERTENSION

The ACCP guidelines for medical management of idiopathic pulmonary artery hypertension in adults recommend routine anticoagulant prophylaxis with VKA, although there is variation with respect to the target range recommended. Extrapolating the adult data, the ACCP guidelines recommend anticoagulant therapy in children with idiopathic pulmonary artery hypertension along with vasodilator or other medical therapy.[13]

## OTHER INDICATIONS

### Prophylaxis after Glenn shunt

Thrombotic complications following the Glenn shunt are infrequently reported.[20] There are no published data supporting the use of oral anticoagulants after Glenn shunt.

### Endovascular stents

There are no reported data regarding the utility of oral anticoagulant therapy to prevent stent thrombosis following endovascular stent therapy for various indications (branch pulmonary artery stenosis, coarctation of aorta, etc.) in children.

### Newer oral anticoagulants

Ximelagatran is a class of new oral anticoagulants acting by direct thrombin inhibition. It is a prodrug of the active site-directed thrombin inhibitor, melagatran. Ximelagatran has a plasma half-life of 4–5 h and is administered orally twice daily. The most common side-effect of ximelagatran is a reversible elevation of liver enzymes. One major advantage of ximelagatran is a predictable anticoagulant response enabling fixed-dose administration without routine coagulation monitoring. Ximelagatran has been evaluated for thromboprophylaxis in high-risk orthopedic patients, treatment of venous TE and prevention of cardioembolic events in patients with nonvalvular atrial fibrillation (SPORTIF III and V trials).[21] There is no data on use of ximelagatran in pediatric patients. Rivaroxaban is another oral anticoagulant that acts by direct inhibition of activated Factor X (FXa).[22] Multiple studies to evaluate its efficacy in adult patients in preventing venous TE are underway.

## CONCLUSIONS

Most of the recommendations regarding the use of oral anticoagulants in children have been extrapolated from the adult literature, with very few randomized trials performed in the pediatric population. Warfarin continues to be the most common agent in the pediatric age group. There is a pressing need for more evidence base regarding the use of these agents in specific pediatric cardiac indications like prophylaxis after Fontan operation and Kawasaki disease with giant coronary aneurysms.
